# The Impact of Social Media Use Interventions on Mental Well-Being: Systematic Review

**DOI:** 10.2196/44922

**Published:** 2023-08-11

**Authors:** Ruth Plackett, Alexandra Blyth, Patricia Schartau

**Affiliations:** 1 Research Department of Primary Care & Population Health University College London London United Kingdom

**Keywords:** review, social media, mental well-being, mental health, addiction, intervention, mobile phone

## Abstract

**Background:**

There is some evidence that more social media use is related to poorer mental well-being and that social media use can become problematic when it starts to interfere with a person’s daily life and mental well-being. To address this issue and improve users’ mental well-being, social media use interventions (eg, abstinence from social media) have been developed and evaluated. However, there is limited understanding of the effectiveness of these interventions in improving mental well-being.

**Objective:**

This systematic review aimed to synthesize the literature on the effectiveness of social media use interventions in improving mental well-being in adults.

**Methods:**

A systematic search (January 1, 2004, to July 31, 2022) was completed across 3 databases in accordance with the PRISMA (Preferred Reporting Items for Systematic Reviews and Meta-Analyses) guidelines. Experimental studies evaluating the impact of social media use interventions on mental well-being in adults were included. Outcomes related to mental well-being, such as depression, anxiety, stress, and loneliness, were included. A narrative synthesis without meta-analysis was completed to summarize the study characteristics and effectiveness by outcome and intervention type. The Effective Public Health Practice Project Quality Assessment Tool was used to measure the quality of the studies.

**Results:**

Of the 2785 studies identified through the systematic search, 23 (0.83%) were included in the analysis. Many of the included studies (9/23, 39%) found improvements in mental well-being, some (7/23, 30%) found mixed effects, and others (7/23, 30%) found no effect on mental well-being. Therapy-based interventions that used techniques such as cognitive behavioral therapy were more effective than limiting use of social media or full abstinence from social media, with 83% (5/6) of these studies showing improvements in mental well-being compared with 20% (1/5) and 25% (3/12), respectively. Depression was the most frequently investigated and improved outcome with 70% (7/10) of the studies showing a significant improvement in depression after the intervention, whereas other outcomes showed more varied results. Quality was poor, with 96% (22/23) of the studies receiving a *weak* global score, mostly for issues related to selection bias because most of the studies (16/23, 70%) used a convenience sampling of university students.

**Conclusions:**

This review provides some evidence that social media use interventions are effective in improving mental well-being, especially for depression and when using therapy-based interventions. Further experimental and longitudinal research is needed with representative samples to investigate who may benefit most from social media use interventions. This will help to develop guidance and recommendations for policy makers and clinicians on how best to manage problematic social media use.

## Introduction

### Background

Over the past decade, the rates of poor mental well-being have steadily increased in the United Kingdom, with steep increases seen for young adults [1,2]. As of 2022, in the United Kingdom, 1 in 4 individuals aged 17 to 19 years had reported a probable mental disorder, up from 1 in 10 in 2017 [3]. At the same time, social media use is on the rise, and it is estimated that 4.59 billion people globally used at least 1 form of social media in 2022 [4-6]. *Social media* generally refers to “internet-based tools that allow individuals and communities to gather and communicate; to share information, ideas, personal messages, images, and other content; and, in some cases, to collaborate with other users in real time” [7]. Social media has significantly changed how people communicate, form and maintain relationships, and perceive each other, and there is concern about how this affects mental health [8].

Evidence on the impact of social media on mental health is conflicting [9]. Some studies report benefits of social media use for mental health, including increased social support, strengthened bonds, and help seeking [10,11]. Other evidence has shown that high social media use has been linked with depression, anxiety, psychological problems, and distress, particularly for young people [12,13]. When social media use begins to interfere with everyday life, it can be considered problematic, with the most severe form arguably termed *social media addiction* [4,14]. Problematic social media use is described as a preoccupation with social media, resulting in distraction from primary tasks and the neglect of responsibilities in other aspects of life [15,16]. Reports suggest that 17.4% of social media users are affected by some form of problematic social media use, and it is most prevalent in adolescents and young adults [4,17]. Previous research has identified significant positive associations between problematic social media use and depression and anxiety [18]. However, the quality of studies linking social media use and mental well-being is limited by a reliance on unvalidated self-reported measures to assess social media use and by cross-sectional study designs in which causality cannot be inferred [9]. In addition, much of the research on the relationship between social media use and mental health has focused on adolescents, but there is growing evidence that social media use plays a role in adult mental health, particularly for young adults [18].

### Social Media Use Interventions

Studies have explored the effectiveness of different types of social media use interventions to improve mental well-being, ranging from therapy-based approaches and taking complete breaks from social media to limiting social media use to a few hours a day [19-21]. Therapy-based approaches tend to use therapeutic techniques such as cognitive behavioral therapy (CBT) or group psychological counseling to prompt reflection on behaviors, thoughts, and feelings around social media and consideration of time management; for example, weekly group psychological counseling and CBT diaries have been used to help students manage their social media use by focusing on how they spend their time and how they can improve their relationships and communication skills offline [22]. These types of interventions are thought to bring about behavior change through facilitating self-control and reflection [23]. These therapeutic interventions can help individuals to regulate their social media use and reprioritize their social activity, which may improve well-being [24].

A recent systematic review that explored social media use interventions where participants had time-outs from smartphone use, or what is termed a *digital detox*, found mixed impacts of these interventions on mental well-being [25]. However, the review did not distinguish between abstinence from smartphone use more generally and specific abstinence from social media and did not explore effectiveness by the different types of social media interventions, such as limited use or full abstinence. Therefore, it is unclear what the effects of different types of interventions are on social media use and on mental well-being. It is also unclear from the literature what specific effects social media use interventions have on adults because much of the previous research in this area has focused on adolescents. Young adults are of particular interest because they have been identified as being vulnerable to problematic social media use in previous research [17,26]. A review specifically synthesizing the evidence on the effectiveness of social media use interventions on adults’ mental well-being will help to identify how best to support those with problematic social media use and poor mental well-being. Synthesizing these experimental studies will also help to understand the relationship between social media use and mental well-being. This systematic review aimed to (1) identify and describe evaluated social media use interventions, (2) report the effectiveness of these interventions on mental well-being outcomes, and (3) evaluate the quality of current research.

## Methods

This review was completed in accordance with the PRISMA (Preferred Reporting Items for Systematic Reviews and Meta-Analyses) guidelines [27], and the protocol is available via the Open Science Framework [28].

### Search Strategy

The search was limited to studies published between January 1, 2004 (because the year 2004 marked the advent of widespread use of social media platforms), and July 31, 2022 [5]. The search strategy was developed by the research team with input from an experienced librarian. Three electronic databases were searched independently: MEDLINE, PsycINFO, and Web of Science. Papers at full-text screening were used for backward citation chaining, and the reference lists of similar previous reviews were checked for additional references. The search strategy for MEDLINE can be found in Multimedia Appendix 1.

### Inclusion and Exclusion Criteria

The inclusion and exclusion criteria listed in Table 1 were applied.

**Table 1:** Inclusion and exclusion criteria.

Concepts	Inclusion criteria	Exclusion criteria
Population	Adults (age ≥18 years) All countries and any sex	Age <18 years
Intervention	Interventions that explicitly aim to reduce social media use through behavioral methods (eg, limiting social media access) or therapy-based methods (eg, CBT^a^)	Interventions that target general internet use, communication methods (eg, texting), and smartphone use
Comparator	Treatment as usual No intervention Pre- and postintervention comparison	N/A^b^
Outcome	Subjective and objective mental well-being or mental health measures (eg, self-reported measures). Measures related to mental well-being or those related to factors that may inhibit well-being (eg, stress and loneliness) were also included to encompass the broad definition of mental well-being as a state in which a person can realize their own abilities, cope with the stresses of life, and contribute to their community [29]. FOMO^c^ was also included because it has been found to be associated with problematic social media use and poorer mental well-being [30-32]	Outcomes regarding time spent on social media or type of social media use
Study types	Randomized controlled trials Quasi-experimental designs Pre-post studies	Cross-sectional studies Qualitative studies
Publication types	Peer-reviewed articles Full text available	Conference papers, editorial letters, meeting abstracts, gray literature, theses, and systematic reviews Articles not in English

^a^CBT: cognitive behavioral therapy.

^b^N/A: not applicable.

^c^FOMO: fear of missing out.

### Screening

We used the referencing manager software Rayyan to screen articles. Titles and abstracts were screened based on the inclusion and exclusion criteria, and 10.02% (279/2785) of the abstracts were screened by a second reviewer, with any conflicts resolved in discussion. The Cohen κ score was 0.56, with moderate agreement [33]. The full texts of the remaining articles were then screened, with 10% (4/42) screened by a second reviewer.

### Data Extraction

Information on the authors, year, country of origin, aims, methods, types of interventions, main findings, and limitations of each study was extracted using a data extraction table in Excel (Microsoft Corp). The extraction of 26% (11/42) of the full-text articles was checked by a second reviewer to ensure accuracy and consistency.

### Quality Assessment

The Effective Public Health Practice Project Quality Assessment Tool was used to assess quality because this is a validated tool designed to assess quality in public health topics [34]. All studies were given a global score (strong, moderate, or weak) based on 6 key topics: selection bias, study design, confounders, blinding, data collection method, and withdrawals or dropouts. Refer to Multimedia Appendix 2 for a breakdown of the scoring criteria for each key area and overall.

### Analysis

A narrative synthesis without meta-analysis was completed owing to the heterogeneity of the outcomes and interventions. We summarized the studies, intervention characteristics, and effectiveness by outcome and type of intervention. We calculated the effect size (Cohen *d*) for all studies, where possible, to compare effectiveness across outcomes and intervention types.

## Results

### Search Results

**Figure 1:**
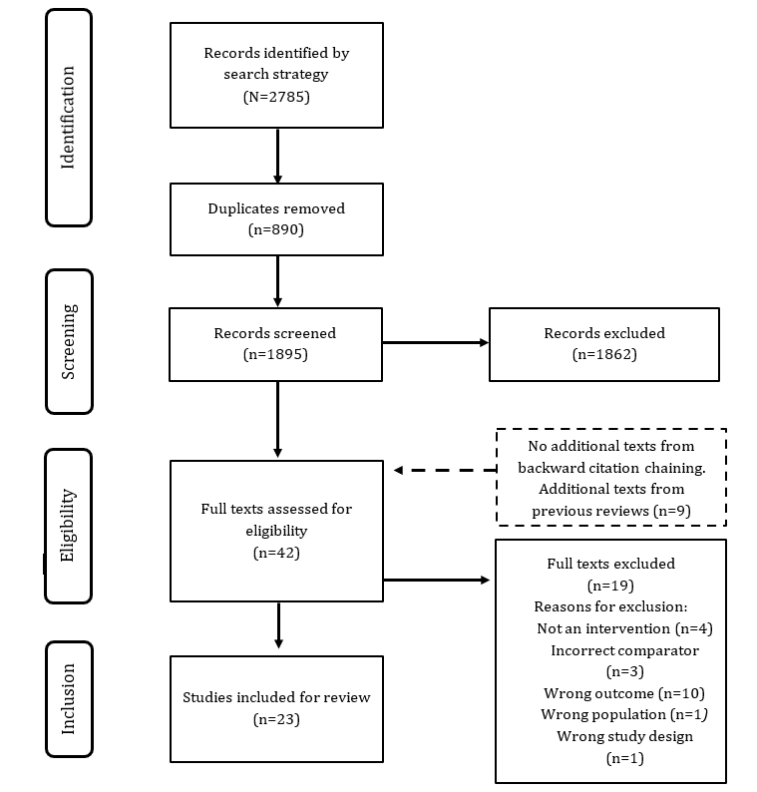
PRISMA (Preferred Reporting Items for Systematic Reviews and Meta-Analyses) flowchart of the systematic search results.

The details of the search process and included studies are summarized in Figure 1.

The search strategy yielded 2785 research papers, of which, after removing duplicates, 1895 (68.05%) were selected for title and abstract screening. Of these 1895 papers, we excluded 1862 (98.26%) based on the inclusion and exclusion criteria and added 9 texts based on the reference list of a previous review. Of these 42 papers eligible for full-text screening, 23 (55%) were included for final analysis.

### Study Characteristics

Study characteristics are summarized in Table 2. All studies were published between 2016 and 2022, with the most common locations being the United States (6/23, 26%), the United Kingdom (5/23, 22%), and China (3/23, 13%). Most of the interventions targeted social media use on general social media sites (10/23, 44%) and Facebook (10/23, 44%), followed by those targeting Instagram (6/23, 26%), Twitter (3/23, 13%), Snapchat (3/23, 13%), TikTok (2/23, 9%), Pinterest (1/23, 4%), and Tumblr (1/23, 4%). Almost a third (7/23, 30%) of the studies targeted the use of multiple specific social media platforms. Randomized controlled trials were the most frequent study design (21/23, 91%). Only 2 (9%) of the 23 studies were pre- and postintervention studies, measuring mental well-being before and after an intervention with no control group [35,36]. There were 3 main types of interventions. Most of the studies (12/23, 52%) evaluated the impact of full abstinence from social media, with the abstinence period ranging from 1 day to 4 weeks. This was followed by therapy-based interventions such as counseling or CBT approaches (6/23, 26%) [22]. Many of the therapy-based types of interventions were self-guided (4/6, 67%) [37-40], with 2 (50%) of these 4 interventions using internet-based app–based platforms [38,39] and 2 (50%) of these 4 interventions using CBT-based diaries to reflect on social media use and time management [37,40]. Of the 6 therapy-based interventions, 1 (17%) used a mixture of in-person peer-based group psychological counseling and an internet-based social media group to share suggestions for alternative activities to social media use [22], 1 (17%) used entirely in-person sessions with training in basic mindfulness skills and values clarification based on acceptance and commitment therapy concepts [35], and 1 (17%) also encouraged limitation of social media use to only a few hours a day [37]. Roughly a quarter (5/23, 22%) of all included studies explored the effect of limiting social media use per day, with intervals ranging from 10- to 60-minute restrictions. More than a third (9/23, 39%) of the included interventions lasted 1 week, with the shortest lasting 1 day and the longest lasting 5 weeks [35].

**Table 2:** Characteristics of studies and social media use interventions sorted by intervention type.

Study, year; country		Study design	Intervention type	Social media intervention target	Intervention duration	Comparator	Sample size, n	Age (years), range (mean, SD)
**Interventions involving full abstinence from social media**								
	Brown and Kuss [36], 2020; United Kingdom	Pre-post study	Full abstinence	General^a^	1 week	None	61	20-49 (24.40, 4.95)
	Fioravanti et al [41], 2020; Italy	RCT^b^	Full abstinence	Instagram	1 week	Control^c^	80; IG^d^: 40, CG^e^: 40	≥18 (25.05, 4.17)
	Hall et al [42], 2019; United States	RCT	Full abstinence	Facebook, Instagram, Snapchat, and Twitter	4 groups: 1 week, 2 weeks, 3 weeks, and 4 weeks	Control	130; IG 1 week: 28, IG 2 weeks: 17, IG 3 weeks: 24, and IG 4 weeks: 26; CG: 35	18-68 (26.80, 11.40)
	Hanley et al [43], 2019; Australia	RCT	Full abstinence	Facebook and Instagram	1 week	Control	78; IG: 40, CG: 38	18-48 (30.85, 7.12)
	Lambert et al [44], 2022; United Kingdom	RCT	Full abstinence	Facebook, Twitter, TikTok, and Instagram	1 week	Control	154; IG: 81, CG: 73	≥18 (28.90, 12.75)
	Mitev et al [45], 2021; United Kingdom and Bulgaria	RCT (crossover trial^f^)	Full abstinence	General	3 days	Control	232; IG: 116, CG: 116	18-71 (24.70, N/R^g^)
	Mosquera et al [46], 2020; United States	RCT	Full abstinence	Facebook	1 week	Control	167; IG: 77, CG: 90	N/R; undergraduates
	Przybylski et al [47], 2021; United Kingdom, United States, and Hong Kong	RCT (crossover trial)	Full abstinence	General	1 day	Control	297; IG: 297, CG: 297	18-56 (20.50, 2.86)
	Tromholt [48], 2016; Denmark	RCT	Full abstinence	Facebook	1 week	Control	888; IG: 516, CG: 372	N/R (34.00, 8.74)
	Turel et al [49], 2018; United States	2×2 RCT	Full abstinence	Facebook	1 week	Control	555; IG: 413, CG: 142	19-54 (24.01, 4.14)
	Vally and D’Souza [50], 2019; United Arab Emirates	RCT	Full abstinence	General	1 week	Control	78; IG: 39, CG: 39	18-27 (22.13, N/R)
	Vanman et al [51], 2018; Australia	RCT	Full abstinence	Facebook	5 days	Control	138; IG: 60, CG: 78	18-40 (22.43, N/R)
**Therapy-based interventions**								
	Chen et al [22], 2022; China	RCT	Group psychological counseling	General	1 month	Control	60; IG: 30, CG: 30	N/R; undergraduates
	Hou et al [40], 2019; China	2×2 RCT	Cognitive reconstruction, reminder cards, and diaries	General	2 weeks	Control	38; IG: 21, CG: 17	N/R (19.71, 1.43)
	O’Connell [35], 2020; United Arab Emirates	Pre-post study	1 hour per week mindfulness workshop	General	5 weeks	None	24; N/R	18-25 (N/R)
	Esmaeili Rad and Ahmadi [38], 2018; Iran	RCT	Reality therapy mobile app+reflective questionnaires	General	2 weeks	Control (only questionnaires)	200; IG: 100, CG: 100	18-28 (N/R)
	Throuvala et al [39], 2020; United Kingdom	RCT	CBT^h^-based app	General	10 days	Control	143; IG: 72, CG: 71	18-32 (20.72, 3.12)
	Zhou et al [37], 2021; China	RCT	CBT-based part abstinence (4 of 7 weekdays)+daily reflective diaries	General	2 weeks	Control (social media as usual+daily diaries)	65; IG: 33, CG: 32	N/R (28.80, 4.90)
**Interventions involving limited social media use**								
	Brailovskaia et al [52], 2020; Germany	RCT	Limited use (20 min/d)	Facebook	2 weeks	Control	286; IG: 140; CG: 146	18-59 (25.39, 5.89)
	Graham et al [53], 2021; New Zealand	RCT	Limited use (10 min/d)	Facebook, Instagram, and Snapchat	1 week	Control	184; IG: 92, CG: 92	18-61 (22.46, 6.83)
	Hunt et al [54], 2018; United States	RCT	Limited use (10 min/d)	Facebook, Instagram, and Snapchat	3 weeks	Control	143; N/R	N/R; undergraduates
	Hunt et al [55], 2021; United States	RCT	Limited use (30 min/d)	Facebook, Instagram, Twitter, and Snapchat	3 weeks	(1) Control and (2) limited active group (30 min/d+1 action every 3 min, eg, posting and replying)	88; N/R	N/R; undergraduates
	Thai et al [56], 2021; Canada	RCT	Limited use (60 min/d)	Instagram, Facebook, Twitter, Snapchat, TikTok, Pinterest, and Tumblr	3 weeks	Control	38; IG: 16, CG: 22	17-25 (N/R, 0.94)

^a^Targeting any social media platform.

^b^RCT: randomized controlled trial.

^c^Usual social media use.

^d^IG: intervention group.

^e^CG: control group.

^f^All participants receive all interventions, but the order in which they receive them (the sequence) is randomized.

^g^N/R: not reported.

^h^CBT: cognitive behavioral therapy.

### Sample Characteristics

Sample sizes ranged from 24 to 888 individuals, with 44% (10/23) of the studies including a sample size of <100 people. Where provided, sample ages ranged from 17 to 71 years, and 61% (14/23) of the studies reported mean ages between 20 and 30 years. Of the 23 studies, 4 (17%) did not provide ages and categorized participants as *undergraduates* [22,46,54,55]. A little more than half (12/23, 52%) of the studies recruited participants via university sampling, 30% (7/23) used web advertisements, and 17% (4/23) combined these methods [39,42,51,53].

### Quality Assessment

Nearly all studies (22/23, 96%) were given a *weak* global score, and no studies achieved a *strong* global score. Only the study by Throuvala et al [39] achieved a *moderate* score and this study showed a beneficial effect of the intervention on outcomes. Most of the studies were subject to selection bias, with 70% (16/23) being of moderate quality and 30% (7/23) being of weak quality on this criterion, because most of the studies (16/23, 70%) used convenience sampling from university populations. Most of the studies (21/23, 91%) did not report blinding of the researcher or participants. A little more than a third (8/23, 35%) of the studies were weak in study design because although they randomized participants, they did not report how they did this. The studies had relatively low withdrawals and dropouts, with a little more than half (12/23, 52%) reporting that ≥80% of the participants completed the studies. Less than half (9/23, 39%) of the studies fully accounted for confounding variables. A little more than half (12/23, 52%) demonstrated that the data collection tools used were reliable and valid. Scores for all sections are provided in Multimedia Appendix 3 alongside the effectiveness of the interventions.

### Mental Well-Being Outcomes and Effectiveness

The details of study outcomes in relation to mental well-being are provided in Table 3. The main outcomes related to mental well-being reported in the studies were depression, life satisfaction, anxiety, fear of missing out (FOMO), mental well-being, positive affect, negative affect, loneliness, stress, self-esteem, and mindfulness. Depression was the most common outcome that was assessed (10/23, 44%), followed by life satisfaction (9/23, 39%), mental well-being (8/23, 35%), and anxiety (6/23, 26%). The least common outcomes that were assessed were self-esteem (3/23, 13%) and mindfulness (2/23, 9%). Most of the studies (18/23, 78%) investigated >1 mental well-being outcome. More than a third (9/23, 39%) of the studies demonstrated improvements in well-being–related outcomes. Almost one-third (7/23, 30%) of the studies found mixed effects across different well-being–related outcomes [35,41,46,49-51,54], and almost a third (7/23, 30%) found no effect [42,43,45,47,52,53,56].

**Figure 2:**
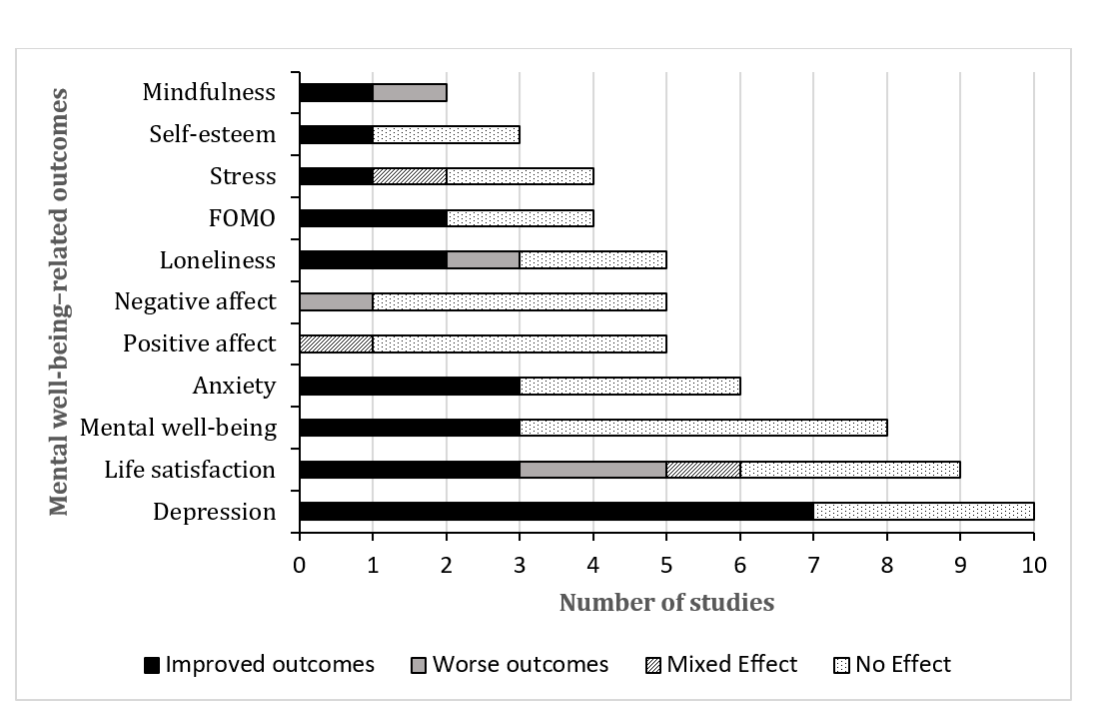
Summary of social media use intervention effects on mental well-being–related outcomes. FOMO: fear of missing out.

An overview of the effectiveness of the interventions by outcome is shown in Figure 2. The most improved outcome was depression, with 70% (7/10) of the studies that measured this outcome showing a benefit of the intervention, with large or medium effect sizes being reported [22,38,44,46,48,54,55], whereas 30% (3/10) showed no effect of the intervention [35,52,56]. Anxiety was the next most improved outcome, with 50% (3/6) of the studies that assessed this outcome showing significant improvement in anxiety, with medium and large effect sizes being reported [38,39,44]; however, 50% (3/6) reported no effect on anxiety [35,54,56]. FOMO also improved in 50% (2/4) of the studies assessing this outcome, with medium and small effect sizes being reported [36,39], whereas 50% (2/4) found no effect on FOMO [35,54]. Brown and Kuss [36] also explored the effect on FOMO based on gender and identified no significant differences between men and women after the intervention. Mental well-being improved in 38% (3/8) of the studies assessing this outcome, with small, medium, and large effect sizes being reported [36,40,44], whereas 63% (5/8) found no significant improvements in mental well-being [35,42,45,53,54]. Self-esteem improved in 33% (1/3) of the studies assessing this outcome, with a medium effect size being reported [40], but 67% (2/3) found no effect of the intervention on self-esteem [47,54].

The other outcomes showed mixed and some negative results. Loneliness was reduced in 40% (2/5) of the studies that measured this outcome [22,54], whereas 40% (2/5) found no effect [42,51]; however, 1 (20%) of these 5 studies also found that the intervention increased loneliness, with a medium effect size being reported [50]. Mindfulness improved in 1 (50%) of the 2 studies that measured this outcome, with a large effect size being reported [39], but it was found to reduce in another study (1/2, 50%), with a small effect size being reported [35]. Life satisfaction improved in a third (3/9, 33%) of the studies that measured this outcome, with small, medium, and large effect sizes being reported [37,38,48], whereas another third (3/9, 33%) of the studies showed no effect [43,46,52], and 22% (2/9) found full abstinence to be harmful, reducing life satisfaction, with medium effect sizes being reported [50,51]. Of these 9 studies, 1 (11%) found mixed effects because a significant improvement in life satisfaction was found for women, with a large effect size being reported, but not for men [41]. For stress, 1 (25%) of the 4 studies that measured this outcome found reductions in stress, with a medium effect size being reported [39], but half (2/4, 50%) of the studies showed no effect of the intervention on stress [50,51], whereas 25% (1/4) showed mixed effects because absolute stress reduced but the relative stress score compared with the score at baseline did not [49]. For negative affect, 1 (20%) of the 5 studies that measured this outcome found that negative affect increased after the intervention, with a small effect size being reported [50], but the other studies (4/5, 80%) showed no effect [41,43,47,51]. Positive affect also did not change after the intervention in most of the studies (4/5, 80%) [43,47,50,51]; however, 20% (1/5) found mixed results because positive affect improved for women, with a small effect size being reported, but not for men [41].

**Table 3:** Effectiveness of social media use interventions on mental well-being sorted by intervention type.

Study, year		Outcomes	Measures	Comparison measurement	Postintervention-reported values (unless labeled)	Effect size (Cohen *d*) and interpretation	Main finding	Direction of effect
**Interventions involving full abstinence from social media**								
	Brown and Kuss [36], 2020	FOMO^a^ Mental well-being	FoMOs^b^ WEMWBS^c^	Mean difference between before and after the intervention (SD)	−3.20 (5.22)^d^ 4.00 (5.97)^d^	0.55 (M^e^) 0.47 (S^f^)	Significant improvements after the intervention	↑^g^
	Fioravanti et al [41], 2020	Life satisfaction Positive affect Negative affect	SWLS^h^ PANAS^i^ PANAS	Calculated mean difference between IG^j^ and CG^k^ for women and men	Women 2.40^l^ 5.45^l^ −0.30 Men 1.90 0.50 −2.25	Women 0.81 (L^m^) 1.09 (L) 0.04 (X^n^) Men 0.36 (S) 0.07 (X) 0.31 (S)	Significantly improved life satisfaction and positive affect for women but not for men in the IG compared with those in the CG	↕^o^
	Hall et al [42], 2019	Mental well-being Loneliness	Four items from the SF-36^p^ A short scale for measuring loneliness in large surveys [57]	Mean (SD)	CG 4.79 (1.31) 2.57 (1.17) IG 4.81 (1.31) 2.57 (1.24)	0.02 (X) 0.00 (X)	No significant difference for the IG compared with the CG across all measures	↔^q^
	Hanley et al [43], 2019	Life satisfaction Positive affect Negative affect	Quality of Life Enjoyment and Satisfaction Questionnaire-18 PANAS PANAS	Standardized coefficients from multiple regression	0.05 −0.16 0.10	0.01 (X) 0.32 (S) 0.20 (S)	No significant difference for the IG compared with the CG across all measures	↔
	Lambert et al [44], 2022	Mental well-being Depression Anxiety	WEMWBS Patient Health Questionnaire depression scale GAD-7^r^	Mean (SD)	CG 45.05 (8.06) 6.95 (4.45) 5.94 (4.30) IG 55.93 (7.65)^d^ 4.84 (3.89)^d^ 3.88 (3.84)^l^	1.38 (L) 0.50 (M) 0.51 (M)	Significant improvements for the IG compared with the CG across all measures	↑
	Mitev et al [45], 2021	Mental well-being	Daily satisfaction question, self-esteem scale, and positive and negative affect scales were combined to create an overall composite score of participants’ well-being	*F* value and partial eta–squared value	*F*_2,416_=0.11; P=.89; η_p_^2^=.001	0.06 (X)	No significant difference for the IG compared with the CG	↔
	Mosquera et al [46], 2020	Life satisfaction Depression	One question taken from the OECDs Better Life Initiative One question taken from the OECD Better Life Initiative	Mean difference between before and after the intervention (SD)	(3.36) −0.57 (2.97)l	—^t^	Significant improvements for the IG compared with the CG for depression but not for life satisfaction	↕
	Przybylski et al [47], 2021	Positive affect Negative affect Self-esteem	PANAS PANAS Rosenburg Self-Esteem Scale (10-item version)	Mean (SD)	CG 2.78 (0.78)^u^ 1.71 (0.69) 3.01 (0.49) IG 2.72 (0.81) 1.81 (0.71) 2.99 (0.50)	0.08 (X) 0.14 (X) 0.04 (X)	No significant difference for the IG compared with the CG	↔
	Tromholt [48], 2016	Life satisfaction Depression or emotion	Life satisfaction questionnaire developed for the investigation Four items from the CES-D^v^ and 4 items from PANAS combined	Mean (SD)	CG 7.74 (1.43) 33.99 (6.81) IG 8.11 (1.23)d 36.21 (6.09)d	0.37 (S) 1.22 (L)	Significant improvements for the IG compared with the CG across all measures	↑
	Turel et al [49], 2018	Absolute stress Relative stress	PSS^w^ PSS	Marginal means (95% CI)	CG 0.50 (0.33 to 0.68) 0.09 (0.04 to 0.15) IG 0.72 (0.62 to 0.82)^l^ 0.14 (0.10 to 0.17)	0.18 (X) 0.13 (X)	Significantly reduced absolute stress in the IG but not relative stress compared with the CG	↕
	Vally and D’Souza [50], 2019	Life satisfaction Positive affect Negative affect Loneliness Stress	SWLS PANAS PANAS Social and Emotional Loneliness Scale for Adults PSS	Mean (SD)	CG 5.16 (1.14) 3.31 (0.81) 2.36 (0.84) 3.08 (1.13) 1.78 (0.51) IG 4.37 (1.26)l 3.34 (0.63) 2.60 (0.89)l 3.81 (1.30)l 1.79 (0.49)	0.66 (M) 0.04 (X) 0.28 (S) 0.60 (M) 0.02 (X)	Significantly reduced life satisfaction and increased negative feelings and loneliness for the IG compared with the CG but no difference in positive affect or stress	↕
	Vanman et al [51], 2018	Life satisfaction Stress Positive affect Negative affect Loneliness	SWLS PSS PANAS PANAS Short form of the Social and Emotional Loneliness Scale for Adults	Mean difference between before and after the intervention (SD)	CG 1.85 (4.97) Means not reported for other outcomes IG −0.93l (5.61) Means not reported for other outcomes	0.54 (M)	The IG had significantly reduced life satisfaction compared with the CG but no other outcomes were significantly different	↕
**Therapy-based interventions**								
	Chen et al [22], 2022	Depression Loneliness	CES-D-20^x^ ULS-8^y^	Mean (SD)	CG 15.70 (9.73) 17.07 (3.52) IG 3.00 (4.65)d 13.17 (3.04)d	1.67 (L) 1.19 (L)	Significant improvements for the IG compared with the CG across all measures	↑
	Hou et al [40], 2019	Self-esteem Mental health and well-being	Chinese version of the Self-Esteem Scale Questionnaire developed from GHQ-30^z^	Mean (SD)	CG 28.35 (3.81) 12.35 (4.58) IG 30.67 (3.17)^d^ 15.71(3.89)^l^	0.66 (M) 0.79 (M)	Significant improvements for the IG compared with the CG across all measures	↑
	O’Connell [35], 2020	Mindfulness FOMO Mental well-being Depression Anxiety	MAAS^aa^ FoMOs PWB^ab^ CES-D Zung Self-Rating Anxiety Scale	Mean difference between before and after the intervention (N/R^ac^)	−0.32l −0.046 −0.052 0.36 0.95	0.32 (S) 0.05 (X) 0.07 (X) 0.03 (X) 0.12 (X)	Significant reduction in mindfulness after the intervention but no difference in FOMO, well-being, depression, or anxiety	↕
	Esmaeili Rad and Ahmadi [38], 2018	Depression Anxiety Life satisfaction	BDI^ad^ BDI SWLS	Mean rank^ae^ (N/R)	CG 50.96 48.74 45.56 IG 42.30d 43.71d 54.72d	0.84 (L) 0.97 (L) 1.04 (L)	Significant improvements within the IG across all measures	↑
	Throuvala et al [39], 2020	Mindfulness Stress Anxiety FOMO	MAAS PSS GAD-7 FoMOs	Mean (SD)	CG 3.37 (0.76) 27.94 (5.24) 17.44 (4.42) 3.32 (1.22) IG 3.97 (0.69)^d^ 24.10 (4.63)^d^ 14.75 (4.43)^d^ 2.86 (1.16)^d^	0.82 (L) 0.77 (M) 0.60 (M) 0.39 (S)	Significant improvements for the IG compared with the CG across all measures	↑
	Zhou et al [37], 2021	Life satisfaction	SWLS	Mean (SD)	CG 4.37 (1.06) IG 4.90 (1.04)l	0.50 (M)	Significant improvements for the IG compared with the CG	↑
**Interventions involving limited social media use**								
	Brailovskaia et al [52], 2020	Life satisfaction Depression	SWLS DASS-21af	Mean difference (95% CI) between the groups	−0.57 (−1.95 to 0.81) 0.15 (−0.90 to 1.20)	0.10 (X) 0.03 (X)	No significant improvement in the IG compared with the CG across both measures	↔
	Graham et al [53], 2021	Well-being	WEMWBS	Mean (SD)	CG 3.43 (0.72) IG 3.51 (0.64)	0.01 (X)	No significant improvement in the IG compared with the CG	↔
	Hunt et al [54], 2018	FOMO Loneliness Anxiety High depression Low depression Self-esteem Mental well-being	FoMOs ULS-8 Spielberger State Anxiety Inventory BDI BDI Rosenberg Self-Esteem Scale PWB	Mean	CG N/R N/R N/R 22.83 4.67 N/R N/R IG N/R N/Rl N/R 14.50l 4.10l N/R N/R	—	Significant improvements in depression and loneliness for the IG compared with the CG; no significance for other outcomes	↕
	Hunt et al [55], 2021	Depression	BDI	Mean (SD)	CG 28.63 (5.04) AGag 16.29 (5.22) IG 14.80 (5.41)l	—	Significant improvements for IG participants who were highly depressed compared with CG and AG participants	↑
	Thai et al [56], 2021	Anxiety Depression	GAD-7 Revised CES-D	Mean (SD)	CG N/R N/R IG 8.54 (4.27) N/R	0.62 (M) 0.57 (M)	No significant improvement in the IG compared with the CG across both measures	↔

^a^FOMO: fear of missing out.

^b^FoMOs: Fear of Missing Out Scale.

^c^WEMWBS: Warwick-Edinburgh Mental Well-Being Scale.

^d^P<.001.

^e^M: medium.

^f^S: small.

^g^Beneficial effect.

^h^SWLS: Satisfaction With Life Scale.

^i^PANAS: Positive and Negative Affect Schedule.

^j^IG: intervention group.

^k^CG: control group.

^l^P<.05.

^m^L: Large.

^n^X: negligible.

^o^Mixed effects.

^p^SF-36: 36-item Short Form Health Survey.

^q^No effect.

^r^GAD-7: General Anxiety Disorder-7.

^s^OECD: Organisation for Economic Co-operation and Development.

^t^Not available (number in each group was not specified to calculate effect size).

^u^Average mean and SD reported across the 3 countries because the relationship among variables was the same across the countries. Estimates based on unadjusted means because the adjusted means were not provided.

^v^CES-D: Center for Epidemiological Studies Depression Scale.

^w^PSS: Perceived Stress Scale.

^x^CES-D-20: Center for Epidemiological Studies Depression Scale, 20-item version.

^y^ULS-8: University of California Los Angeles Loneliness Scale.

^z^GHQ-30: General Health Questionnaire-30.

^aa^MAAS: Mindful Attention Awareness Scale.

^ab^PWB: Psychological Well-Being Scale.

^ac^N/R: not reported.

^ad^BDI: Beck Depression Inventory.

^ae^Comparing the IG between before the intervention and after. The effect of the intervention between the IG and the CG was not reported and could not be calculated.

^af^DASS-21: Depression Anxiety Stress Scales 21.

^ag^AG: active group (limited use of social media at 30 minutes per day plus 1 action every 3 minutes, eg, posting and replying).

### Effectiveness by Intervention Type

**Figure 3:**
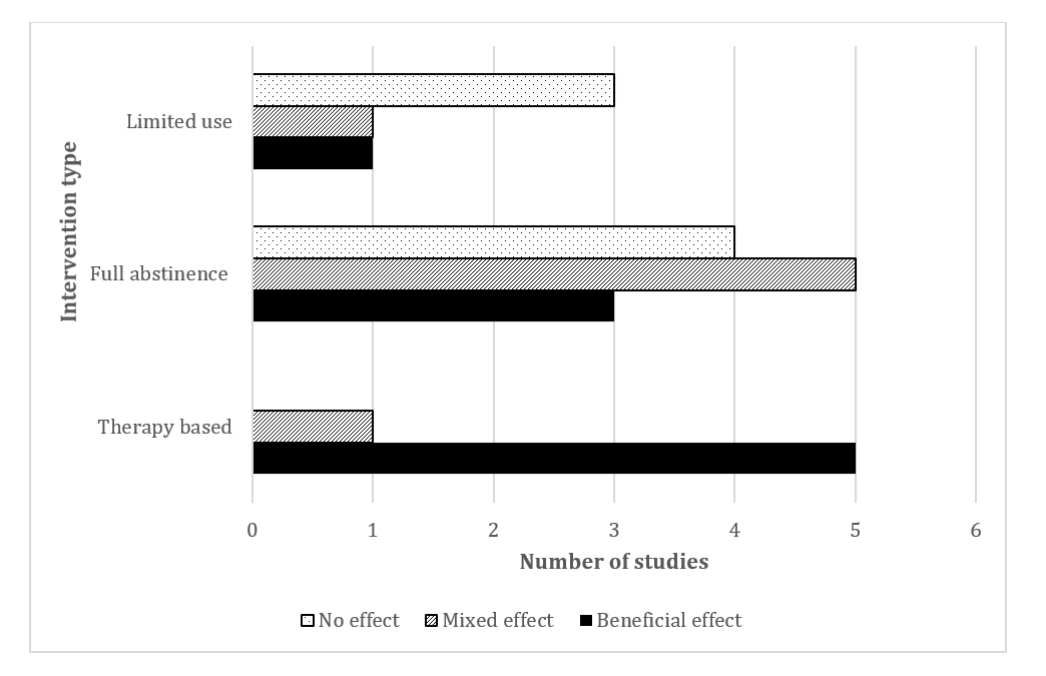
Summary of the effectiveness of the different types of social media use interventions on mental well-being–related outcomes.

Figure 3 shows an overview of the effectiveness of social media use interventions by intervention type. Therapy-based trials were the most effective because 83% (5/6) of the studies that assessed this intervention type found significant improvements in mental well-being outcomes [22,37-40], and only 17% (1/6) found mixed effects [35]. Full-abstinence interventions showed mixed effectiveness overall, with 42% (5/12) of them showing mixed effects [41,46,49-51], a third (4/12, 33%) showing no effect [42,43,45,47], and a quarter (3/12, 25%) showing a benefit of the intervention [36,44,48]. Social media use interventions that limited use also showed mixed effectiveness overall, with more than half (3/5, 60%) of the studies showing no effect [52,53,56] and 20% (1/5) showing an improvement [55] and mixed effects [54], respectively.

## Discussion

### Principal Findings

This review provides some evidence for the improvement of mental well-being in adults after social media use interventions. Of the 23 studies included in our analysis, 9 (39%) demonstrated improvements in mental well-being–related outcomes, with most of them (8/9, 89%) showing medium to large effect sizes. Depression was the most improved outcome overall, with 70% (7/10) of the studies that assessed depression showing improvement. Therapy-based interventions were the most effective, with 83% (5/6) of the studies evaluating these interventions showing improvement. Most of the studies (22/23, 96%) were of low quality, with significant issues related to selection bias and blinding affecting their quality.

This review found that interventions that simply restrict social media use or impose full abstinence may not have as much benefit for mental well-being as therapy-based interventions. These interventions used established approaches such as counseling and CBT-based techniques to encourage mindfulness and reflection on how social media activity affects thoughts, emotions, and behavior. Therapy-based interventions may be more effective in causing behavior change in users than abstinence by enabling them to replace negative actions with structured goals and positive thinking and by providing motivation [58]. Therapy-based interventions may also help to reduce FOMO by encouraging individuals to reevaluate life priorities, focus on other activities, and reduce social comparison and envy [36,39].

We found limiting social media use to be the least effective method. One hypothesis is that users would still have been exposed to social media during the trial and may have intensified their use owing to an awareness of a time restriction. This would have offset any potential positive improvement. Adherence to limiting social media use or abstaining from social media use may also have been challenging for participants, and adherence is difficult to track and measure across devices used to access social media [37]. In 1 study, 19.4% (35/180) of the participants were excluded from analysis because they were unable to abstain from social media for >2 days, which may give an indication of compliance rates in these studies [42]. These findings overall suggest that health and care professionals, mental health charities, and public health bodies should encourage the use of therapy-based approaches to manage social media use rather than focusing on reducing time spent on social media. These interventions can also be relatively cost-effective because this review showed improvements in participants’ well-being after they used internet-based self-guided therapy-based interventions to manage problematic social media use. However, delivering therapy-based approaches to manage (problematic) social media use is currently limited owing to resource and time constraints in health and care systems.

This review found that 3 (13%) of the 23 studies showed a reduction in some mental well-being–related outcomes after the intervention, such as life satisfaction and loneliness [35,50,51]. The causes for these findings could be due to methodological reasons because the authors of 1 (33%) of these 3 studies proposed that their study participants were unaware at the time of recruitment that abstinence from social media may be required [50], which may have made participants less receptive to the intervention and eliminated the beneficial consequences of abstinence that may have arisen in other studies. Previous research also suggests that reducing or limiting social media use can reduce mental well-being by causing a loss of social connection and increasing loneliness [9]. Some individuals are reported to find social connections easier to maintain over the web, with social media enabling users to preserve their relationships [59,60]. Social media can also help to create and maintain social capital, fostering inclusion within web-based communities [61]; for instance, members of the lesbian, gay, bisexual, transgender, queer, and similar minority community report greater levels of social support over the web [62].

This review supports evidence from previous studies that show that the link between social media use and mental well-being is conflicting, with there being some benefits and some disadvantages of social media use related to mental well-being [9,25]. The variation in effectiveness across the studies could be due to individual differences [63]. Different people will have different responses to social media, and self-regulatory capabilities may be affected by factors such as gender, age, and personality traits [64]. Previous research has shown that those with neurotic or introverted tendencies have a higher risk of addiction to internet content [65]. Others may not be affected by social media use owing to elevated levels of digital resilience. Digital resilience is a person’s ability to cope with the negative consequences of being over the web, such as cyberbullying and misleading information [66,67]. Gender has been found to be a moderating factor in previous studies examining the relationship between social media use and mental well-being, with adolescent girls seeming to experience more negative effects from social media use than adolescent boys [4,68,69]. In this review, we found that 1 (4%) of the 23 studies showed that abstinence increased positive affect and life satisfaction for women but not for men, also suggesting that gender may affect the relationship between social media use and mental well-being [41]. Future research is needed to explore who may be most affected by problematic social media use to enable the development of more targeted interventions to improve mental well-being.

### Limitations

There was a large degree of heterogeneity in the studies reviewed, with several different intervention types and outcome measures used. Therefore, it was not possible to conduct a meta-analysis to provide integrated results on the outcomes of the social media use interventions [70]. A further limitation to this review is that the search strategy may not have retrieved all relevant papers owing to the inclusion of English-language publications only. Our review also did not include unpublished studies; thus, it was not possible to estimate the degree of publication bias. This review also did not explore the impact of the type of social media use, such as passive use or active use, because this was out of the scope of the review, but this could affect mental well-being. *Active* use denotes direct messaging, posting, or responding to content, whereas *passive* use corresponds to scrolling and browsing profiles. Previous literature has suggested that passive use is associated with greater declines in subjective well-being [71,72], but a recent review found that this was not supported across 40 survey-based studies [73]. The review suggested that future studies should explore the characteristics of the content of social media as well as its senders and receivers to understand how different uses of social media affect mental well-being [73]. Understanding this relationship could help to develop more targeted problematic social media use interventions that move beyond simply aiming to reduce time spent on social media by targeting the reduction of specific negative activities or interactions.

A major limitation of the studies included in this review is that the majority (16/23, 70%) relied largely on convenience samples of those who were likely to be interested in reducing their social media use and improving their mental health. In addition, more than half (16/23, 70%) of the studies recruited university students. Therefore, these results must be interpreted with caution because they are not generalizable to all adults and are likely to be more relevant for young adults. Furthermore, none of the studies received a *strong* global score for quality using the Effective Public Health Practice Project Quality Assessment Tool. The sustainability of these interventions is also difficult to establish because most of the interventions (20/23, 87%) lasted <1 month, and the outcomes were assessed immediately after the interventions. Only 2 (9%) of the 23 studies included a longer-term measure. Brailovskaia et al [52] found consistency with their short-term recorded outcomes, with no significant difference in life satisfaction and depression between groups 3 months after the intervention. By contrast, Chen et al [22] found that the significant improvements in depression and loneliness for the intervention group continued to remain at 2 months.

### Conclusions

There is some evidence that social media use interventions are effective in improving mental well-being in adults, especially for depression and when using therapy-based interventions. Current experimental research is of low quality, with issues of selection bias making it difficult to generalize the findings. Further experimental and longitudinal research is needed with representative samples to investigate who may benefit most from social media use interventions. Health and care professionals should be aware of the growing evidence that reducing social media use alone is unlikely to benefit mental well-being. Taking a more therapy-based approach and reflecting on how and why individuals are interacting with social media and managing these behaviors could help to improve mental well-being.

## Appendix

Multimedia Appendix 1 Search strategy used for MEDLINE.

Multimedia Appendix 2 Quality assessment tool for quantitative studies scoring criteria.

Multimedia Appendix 3 Quality scores according to the Effective Public Health Practice Project Quality Assessment Tool and overall effectiveness of social media use interventions on outcomes.

## Abbreviations

CBT: cognitive behavioral therapy
FOMO: fear of missing out
PRISMA: Preferred Reporting Items for Systematic Reviews and Meta-Analyses
