# Supplementary material for: The Impact of Social Media Use Interventions on Mental Well-Being: Systematic Review
Source: J Med Internet Res. 2023 Aug 11;25:e44922. doi: 10.2196/44922 (PMC10457695; doi:10.2196/44922)
Supplement: Multimedia Appendix 1 [file jmir_v25i1e44922_app1.docx]

| Multimedia Appendix 1 **Appendix 1: Search strategy used for MEDLINE.**  This strategy was adapted for other databases used. | | | |
| --- | --- | --- | --- |
| **Social Media** | **Mental Well-Being** | **Study Design** | **Intervention** |
| Social Media/  Social Networking/  Online Social Networking/ or online social network*  (Social* adj2 (networking* or media))  Instagram  Facebook  YouTube  Reddit  WeChat  Snapchat  WhatsApp  TikTok | Mental Health  (Mental adj well*)  Anxiety/  Depression/  Depress*  (mental* adj2 (health or hygiene))  (anxiet* or anxious*)  Stress*  Loneliness/  (Self esteem or self-esteem)  Wellbeing or well-being  Life satisfact*  Affect adj2 positive adj2 negative | Exp Randomized Controlled Trial/  Randomi#ed controlled trial  RCT  Random*  Trial*  Evaluation Study/  Qualitative Research/  Experiment*  Controlled design  Intervention*  (pre?intervention$ or preintervention$ or pre intervention$ or post?intervention$ or postintervention$ or post intervention$)  Survey  Investigat* | exp behavior, addictive/ or exp technology addiction/  Addict*  Excessi*  Restrict*  Quit*  Reduc*  Withdraw*  Abstin* |
