# Supplementary material for: The Impact of Social Media Use Interventions on Mental Well-Being: Systematic Review
Source: J Med Internet Res. 2023 Aug 11;25:e44922. doi: 10.2196/44922 (PMC10457695; doi:10.2196/44922)
Supplement: Multimedia Appendix 2 [file jmir_v25i1e44922_app2.docx]

**Appendix 2**

**Quality assessment tool for quantitative studies scoring criteria**

| Criteria | Strong | Moderate | Weak |
| --- | --- | --- | --- |
| Selection bias | The selected individuals are very likely to be representative of the target population and there is greater than 80% participation. | The selected individuals are at least somewhat likely to be representative of the target population; and there is 60 - 79% participation. ‘Moderate’ may also be assigned if it is not fully described. | The selected individuals are not likely to be representative of the target population; or there is less than 60% participation or selection is not described; and the level of participation is not described. |
| Study Design | Will be assigned to those articles that described RCTs and CCTs. | Will be assigned to those that described a cohort analytic study, a case control study, a cohort design, or an interrupted time series. | Will be assigned to those that used any other method or did not state the method used. |
| Confounders | Will be assigned to those articles that controlled for at least 80% of relevant confounders. | Will be given to those studies that controlled for 60 – 79% of relevant confounders. | Will be assigned when less than 60% of relevant confounders were controlled or control of confounders was not described. |
| Blinding | The outcome assessor is not aware of the intervention status of participants; and the study participants are not aware of the research question. | The outcome assessor is not aware of the intervention status of participants; or the study participants are not aware of the research question; or blinding is not described | The outcome assessor is aware of the intervention status of participants; and the study participants are aware of the research question. |
| Data Collection | The data collection tools have been shown to be valid; and the data collection tools have been shown to be reliable. | The data collection tools have been shown to be valid; and the data collection tools have not been shown to be reliable or reliability is not described. | The data collection tools have not been shown to be valid or both reliability and validity described. |
| Withdrawals and Drop Out | Will be assigned when the follow-up rate is 80% or greater. | Will be assigned when the follow-up rate is 60 – 79%. | Will be assigned when a follow-up rate is less than 60% or if the withdrawals and drop-outs were not described. |
| Global | no WEAK ratings | one WEAK rating | two or more WEAK ratings |
