# Supplementary material for: The Impact of Social Media Use Interventions on Mental Well-Being: Systematic Review
Source: J Med Internet Res. 2023 Aug 11;25:e44922. doi: 10.2196/44922 (PMC10457695; doi:10.2196/44922)
Supplement: Multimedia Appendix 3 [file jmir_v25i1e44922_app3.pdf]

	Brailovskaia et al (2020)	Brown and Kuss (2020)	Chen et al (2022)	Fioravanti et al (2020)	Graham et al (2021)	Hall et al (2019)	Hanley et al (2019)	Hou et al (2019)	Hunt et al (2018)	Hunt et al (2021)	Lambert et al (2022)	Mitev et al (2021)	Mosquera et al (2020)	O'Connell (2020)	Przybylski et al (2021)	Esmaeili Rad & Ahmadi (2018)	Thai et al (2021)	Throuvala et al (2020)	Tromholt (2016)	Turel et al (2018)	Vally & D'Souza (2019)	Vanman et al (2018)	Zhou et al (2021)
Intervention effectiveness	−	↑	↑	↕	−	−	−	↑	↕	↑	↑	−	↕	↕	−	↑	−	↑	↑	↕	↕	↕	↑
Selection bias	M	M	W	M	M	M	M	M	W	W	M	M	W	M	M	W	M	M	M	W	W	M	M
Study design	W	M	S	W	S	S	W	W	W	S	S	S	S	M	S	S	S	S	W	W	S	S	W
Confounders	S	S	M	S	W	W	W	W	W	W	S	W	M	W	W	S	W	S	W	S	S	W	S
Blinding	W	W	W	M	W	W	W	W	W	W	W	W	W	W	W	W	W	S	W	W	W	W	W
Data collection	S	W	W	W	S	W	M	W	S	S	W	S	W	S	S	S	S	S	W	W	S	S	W
Withdrawals and Dropouts	S	M	S	S	M	W	M	S	W	W	S	M	S	S	W	S	S	W	M	W	S	S	S
**Global score**	W	W	W	W	W	W	W	W	W	W	W	W	W	W	W	W	W	M	W	W	W	W	W

W:Weak, M:Moderate, S:Strong

− = no effect ↑ = beneficial Effect ↕ = mixed effects
